# ALKBH5 regulates ovarian cancer growth via demethylating long noncoding RNA PVT1 in ovarian cancer

**DOI:** 10.1111/jcmm.18066

**Published:** 2023-12-14

**Authors:** Lin Li, Jie Chen, Ao Wang, Ke Yi

**Affiliations:** ^1^ Department of Obstetrics and Gynecology, West China Second University Hospital Sichuan University Chengdu Sichuan China; ^2^ The Key Laboratory of Birth Defects and Related Diseases of Women and Children (West China Second University Hospital Sichuan University), Ministry of Education Chengdu Sichuan China

**Keywords:** ALKBH5, chemosensitivity, long noncoding RNA PVT1, N6‐methyladenosine, ovarian cancer

## Abstract

The long noncoding RNA PVT1 is reported to act as an oncogene in several kinds of cancers, especially ovarian cancer (OV). Abnormal levels of *N* ^6^‐methyladenosine, a dynamic and reversible modification, are associated with tumorigenesis and malignancies. Our previous study reported that PVT1 plays critical roles in regulating OV. However, it is still largely unknown how m^6^A modification affects OV via PVT1. In this study, we aimed to investigate the regulation of ALKBH5 by affecting PVT1 in OV. We first found that the PVT1 RNA level was higher in OV cells than in IOSE80 cells, and conversely, the m^6^A modification level of PVT1 was lower in OV cells. By searching the HPA, ALKBH5, which is responsible for PVT1 demethylation, was found to be upregulated in OV tissues versus normal ovarian tissues. ALKBH5 binds to PVT1 RNA, and knockdown of ALKBH5 decreased PVT1 RNA levels. ALKBH5 also increased FOXM1 levels by upregulating PVT1, at least partially. Knockdown of ALKBH5 suppressed OV growth, colony formation, tumour formation and invasion, which were partially reversed by overexpression of PVT1. Moreover, ALKBH5 knockdown decreased FOXM1 levels by regulating PVT1 RNA expression, subsequently increasing the sensitivity to carboplatin, 5‐FU and docetaxel chemotherapy. Taken together, these results indicate that ALKBH5 directly regulates the m^6^A modification and stability of PVT1. Then, modified PVT1 further regulates FOXM1 and thus affects malignant behaviours and chemosensitivity in OV cells. All these results indicate that ALKBH5 regulates the malignant behaviour of OV by regulating PVT1/FOXM1.

## 1 INTRODUCTION

Ovarian cancer (OC) is the seventh most common cancer in the world and the fifth leading cause of cancer‐related death in women.^1^ Recent studies have shown that many factors are associated with an increased risk of ovarian cancer; these include early menarche age, no or low parity, hormone replacement therapy (HRT) for menopause, high fat intake and high body mass index (BMI).^2^ Ovarian cancer is divided into three categories according to the type of cell source: epithelial, stromal and germ cell tumours. Among them, epithelial ovarian cancer (EOC) accounts for 90%–95% of malignant ovarian tumours.^3^ Due to the asymptomatic progression and early peritoneal dissemination, ovarian cancer is often diagnosed in its terminal stage, at which point it cannot be cured, resulting in a low survival rate of patients with ovarian cancer.

Noncoding RNAs longer than 200 nt have been named long noncoding RNAs (lncRNAs). LncRNAs can help regulate gene expression at the epigenetic, transcriptional and posttranscriptional levels and participate in biological, physiological and pathological processes through different molecular mechanisms. For example, Linc00858 inhibits the autophagy and apoptosis of colon cancer cells by promoting DNA methylation in the promoter regions of WNK lysine‐deficient protein kinase 2 (WNK2).^4^ LncPXN‐AS1 is spliced into two transcripts, PXN‐AS1‐L and PXN‐AS1‐S, with the action of muscle blind‐like splicing regulator 3 (MBNL3). PXN‐AS1‐S can competitively bind to miRNA‐24 in the 3′ untranslated region (UTR) of proto‐oncogene Paxillin (PXN) mRNA, which protects PXN mRNA from degradation by miRNA‐24, promoting the occurrence of hepatocellular carcinoma.^5^ In the occurrence and progression of tumours, lncRNAs are usually used as gene expression regulators, causing abnormal expression of downstream target genes.

LncRNA PVT1 is encoded by the PVT1 gene on chr8q24.21, which contains 1716 nucleotides. A growing body of research has shown that lncRNA PVT1 has different degrees of abnormal expression in gynaecological malignancies and may affect a variety of tumour cell regulatory processes, which are closely related to the occurrence and progression of tumours. It has been found that lncRNA PVT1 is involved in tumour progression through interaction with miR‐195‐5p in endometrial cancer.^6^ In addition, lncRNA PVT1 increases the methylation level of H3K27me3 in the miR‐195 promoter region by binding EZH2, decreases the expression of miR‐195, and affects EMT processing, thus regulating the reaction of cervical cancer cells to PTX.^7^ LncRNA PVT1 is highly expressed in epithelial ovarian cancer tissues, inhibits the expression of miR‐214 by binding EZH2 and promotes the progression of ovarian cancer.^8^

N6 methyladenosine (m^6^A) is the most common mRNA internal modification in higher eukaryotes^9^, ^10^ and plays an important role in mRNA splicing, miRNA processing and maturation, lncRNA‐mediated transcription inhibition and other RNA metabolism processes.^11^, ^12^ METTL3, METTL14 and WTAP form the m^6^A methyltransferase complex,^13^, ^14^, ^15^ in which METTL3 is the catalytic core enzyme. Studies have shown that an overall high level of METTL3 is an independent factor related to survival in patients with endometrioid epithelial ovarian cancer,^16^ which is closely related to the progression of ovarian cancer. ALKBH5 has been reported to possess demethylation activity and is localized in the nucleus. Silencing or overexpression of ALKBH5 can change m^6^A levels.^17^ ALKBH5 plays an important role in the progression of ovarian cancer. Jiang et al.^18^ found that the high expression of Toll‐like receptor (TLR4) activates the NF‐κB pathway and upregulates ALKBH5, and the expression of NANOG increases after mRNA demethylation, thus enhancing the invasiveness of ovarian cancer cells.

In our previous study, it was demonstrated that PVT1 promotes malignant behaviour and induces chemoresistance in ovarian cancer by epigenetic and posttranscriptional regulation of FOXM1.^19^ To evaluate the effects of m^6^A modification on ovarian cancer by modifying PVT1, our results showed that ALKBH5 demethylated PVT1 and thus stabilized PVT1 and at least partially regulated FOXM1. This study provides more evidence on the regulation of PVT1 by m^6^A modification, which may aid in the development of effective therapeutic strategies for ovarian cancer.

## 2 MATERIALS AND METHODS

### 2.1 Clinical tissue samples

New specimens, comprising of 20 cancerous and 20 neighbouring normal tissues, were procured from ovarian cancer patients who had undergone surgery between March and July 2021 at West China Second Hospital. Prior to the initiation of the study, informed consent was obtained from all participants by adhering to the Declaration of Helsinki and with approval from the Institute Ethics Committee and West China Second Hospital. The samples were gathered and preserved at −80°C until further use.

### 2.2 Cell culture

The Human ovarian cancer cell lines SkOV3 and ES2, and normal ovarian cell line IOS80 are frozen in liquid nitrogen of our laboratory. They were cultured in DMEM (Life Technology) supplemented with 10% fetal bovine serum (FBS), 100‐units/ml penicillin and 100 μg/mL streptomycin (Life Technology). Cells were kept at 37°C in 5% CO_2_ and passaged every 2–3 days.

### 2.3 Global m^6^A/m measurements

To measure total m^6^A methylation level in total RNA, the EpiQuik m^6^A/m RNA Methylation Quantification Kit (Epigentek Group, Farmingdale, NY) was employed followed by the manufacturer's instruction. Briefly, 500 ng of total RNA was involved in each reaction. Labelled total RNA was analysed using LC–MS/MS.

### 2.4 Western blot

Each loading utilized 20 μg of total protein. The proteins were fractionated using SDS‐PAGE and blots were then transferred to a preblocked PVDF membrane (Millipore) containing 5% powdered milk and 5% BSA in PBS with 0.3% Tween 20 at room temperature for 30 min. Following this, the transferred membrane was incubated overnight with primary antibodies, including ALKBH5, beta‐actin, FTO and FOXM1, which were all diluted in ice‐cold PBS at a ratio of 1:1000. After being washed three times with ice‐cold PBS supplemented with 0.2% Tween 20, the blotted membrane was incubated with horseradish peroxidase (HRP)‐labelled secondary antibody for 1 h at room temperature. Finally, the membrane was detected using ECL detection systems (Thermo Scientific, Waltham, MA, USA).

### 2.5 RT‐qPCR

TRIZol (Life technology, Grand Island, NY, USA) was used to extract the Total RNA. For the synthesis of the first‐strand complementary DNA (cDNA), the First strand synthesis Kit (RIBOBIO, Guangzhou, China) was employed with 1 μg of total RNA. The measurement was carried out using Applied Biosystems 7500 Real‐time system (ABI 7500HT instrument), and qPCR was performed using SYBR Green Master Mix (Life Technology, Grand Island, NY, USA). The primers utilized in this research were purchased from RIBOBIO and include PVT1 (forward 5′‐ TGGTGTTCCCCTTTTACTGC‐3′, reverse 5′‐ TGGTGAAACCCCGTCTCTAC‐3′), FOXM1 (Cat. No.: HQP005712) and β‐actin (Cat. No.: HQP108762).

### 2.6 GEPIA and human protein atlas analysis

We utilized GEPIA (Gene Expression Profiling Interactive Analysis), an interactive online server that applies a standardized processing pipeline to analyse RNA sequencing expression data of 9736 tumours and 8587 normal samples from The Cancer Genome Atlas (TCGA) and the Genotype‐Tissue Expression (GTEx) projects, to investigate ALKBH5 mRNA expression levels in OV tissues compared to normal tissues. Additionally, we validated the immunohistochemistry of ALKBH5 in OV tissues using the HPA (https://www.proteinatlas.org/), an open access database that allows scientists in both industry and academia to freely access data for exploring the human proteome. The HPA images related to ALKBH5 in tumour can be found at: https://www.proteinatlas.org/ENSG00000091542‐ALKBH5/pathology/ovarian+cancer#img and https://www.proteinatlas.org/ENSG00000091542‐ALKBH5/pathology/ovarian+cancer#img.

### 2.7 RNA immunoprecipitation assay (RIP)

To evaluate the binding of ALKBH5 to target RNA, RIP assay was performed using a Magna RNA‐binding protein immunoprecipitation kit (Millipore) following its manufacuter's instruction. 2 μg ALKBH5, FTO or Rabbit IgG control antibodies were used for RIP assay. Coprecipitated RNAs were then detected by qRT‐PCR.

### 2.8 Knockdown and overexpression of ALKBH5 or PVT1

Lentivirus expressing scrambled, ALKBH5 or PVT1 shRNAs was purchased from GenePharma Company (Guangzhou, China). In the case of knockdown experiments, cells were infected these lentiviral particles and selected with 3 μg/mL puromycin for 5 days, and stably transfected colonies were collected. In the case of overexpression experiments, lentivirus expressing empty vector, ALKBH5 or PVT1 coding sequence were employed to infecte target cells followed by selection with 3 μg/mL puromycin for 5 day. (GenePharma Company), and then stably expressing colonies were collected.

### 2.9 Cell viability analysis

Cells were harvested at a concentration of 1 × 10^6^ cells/mL and 5000 cells per well were seeded into a 96‐well plate. After an overnight attachment, the prepared solution of Cell Counting Kit‐8 (CCK‐8, Sigma‐Aldrich, St. Louis, MO, USA) was added to each well and incubated for 2 h at 37°C in a dark environment. The cell viability was determined by detecting A450 using a microplate reader (Synergy 2 Multi‐Mode Microplate Reader; BioTek, Winooski, VT, USA) after 24 h.

### 2.10 Cell cycle distribution assay

In order to determine the distribution of the cell cycle, cells were first collected at a concentration of 1 × 10^6^ cells/mL. The cells were then washed three times with ice‐cold PBS and suspended in 70% ethanol at 4°C overnight. Afterward, the cells were pelleted and washed another three times with ice‐cold PBS. Next, the cells were incubated with propidium iodide (PI) at a concentration of 5 μg/mL for 10 min, while being kept away from light. Subsequently, the stained cells were analysed using three‐laser Navios flow cytometers manufactured by Beckman Coulter located in Brea, CA, USA, without undergoing any additional washing steps.

### 2.11 Cell apoptosis assay

The method used to collect cells involved trypsin digestion in the absence of EDTA. Apoptosis was measured using flow cytometry and the Annexin V‐FITC Apoptosis Detection Kit from Bestbio, Shanghai, China, following the manufacturer's protocol. The apoptosis rate (%) was calculated by dividing the total number of apoptotic cells by the total number of cells and then multiplying it by 100. The experiments were repeated at least three times to ensure accuracy and consistency of results.

### 2.12 Migration and invasion

The experiment described in the prompt involved seeding suspended cells in an upper chamber with an 8‐μm pore size, precoated with Matrigel. After 48 h, the cells on the lower face of the chamber were fixed using 4% paraformaldehyde for 10 min at room temperature. Then, they were stained with 0.25% crystal violet from Sigma‐Aldrich. Finally, five views of each chamber were imaged using a X71 (U‐RFL‐T) fluorescence microscope from Olympus in Melville, NY.

### 2.13 Colony formation

To perform the experiment described in the prompt, suspended cells were washed with ice‐cold PBS and adjusted to a concentration of 1 × 106 cells/mL. Then, 5000 cells were plated into each well of a 6‐well plate and cultured for 14 days until colonies were visible to the naked eye. Once visible, the plate was washed three times with ice‐cold PBS. The colonies were fixed using 4% paraformaldehyde for 10 min and stained with 0.25% crystal violet for 20 min. Finally, each well was imaged.

### 2.14 Tumour formation

The cells in suspension were rinsed with ice‐cold PBS and standardized to a concentration of 1 × 106 cells/mL. Next, 5000 cells were placed on a layer of solidified agar (0.6%) in RPMI‐1640 medium containing 10% FBS in six‐well plates. The plates were then incubated at a temperature of 37°C for 2 weeks. Finally, colonies that consisted of 50 or more cells were counted using an Olympus X71 (U‐RFL‐T) fluorescence microscope located in Melville, NY.

### 2.15 Tumour xenografts in nude mice

Ten 6‐week‐old female BALB/c nude mice (weighing 18–20 g) were purchased from The Sichuan Dashuo Laboratory Animal Center in Chengdu, Sichuan. All mice were housed in a temperature‐controlled pathogen‐free environment at 21°C with a 12‐h light–dark cycle following the guidelines outlined in the Guide for the Care and Use of Laboratory Animals. These mice were randomly divided into two groups to receive tumour xenografts. Subsequently, 2 × 10^6^ cells suspended in 100 μL of 1 × PBS were subcutaneously injected into the flank of each mouse. Tumour volume was recorded every 5 days starting from 10 days after injection, using the formula: Tumour volume (mm^3^) = (length × width^2^)/2. After 30 days, the mice were humanely euthanized via cervical dislocation while under anaesthesia, using 1% pentobarbital sodium at a dose of 50 mg/kg through intraperitoneal injection. Half of the tumours were collected and stored for pathological analysis, while the remaining half were harvested for RNA extraction. All animal procedures were performed following the guidelines set out by the National Institutes of Health Guide for the Care and Use of Laboratory Animals and were approved by the Ethics Committee of West China Second Hospital.

### 2.16 Statistical analysis

The study utilized SPSS software version 19.0, developed by SPSS in Chicago, IL, USA, for statistical analysis. The data is expressed as the mean ± standard deviation, and probabilities less than 5% (*p* < 0.05) were deemed statistically significant. All experiments were conducted three or more times to ensure accuracy and consistency.

## 3 RESULTS

### 3.1 PVT1 m^6^A methylation level in OV tissues were lower compared with that in adjacent tissues

**FIGURE 1:**
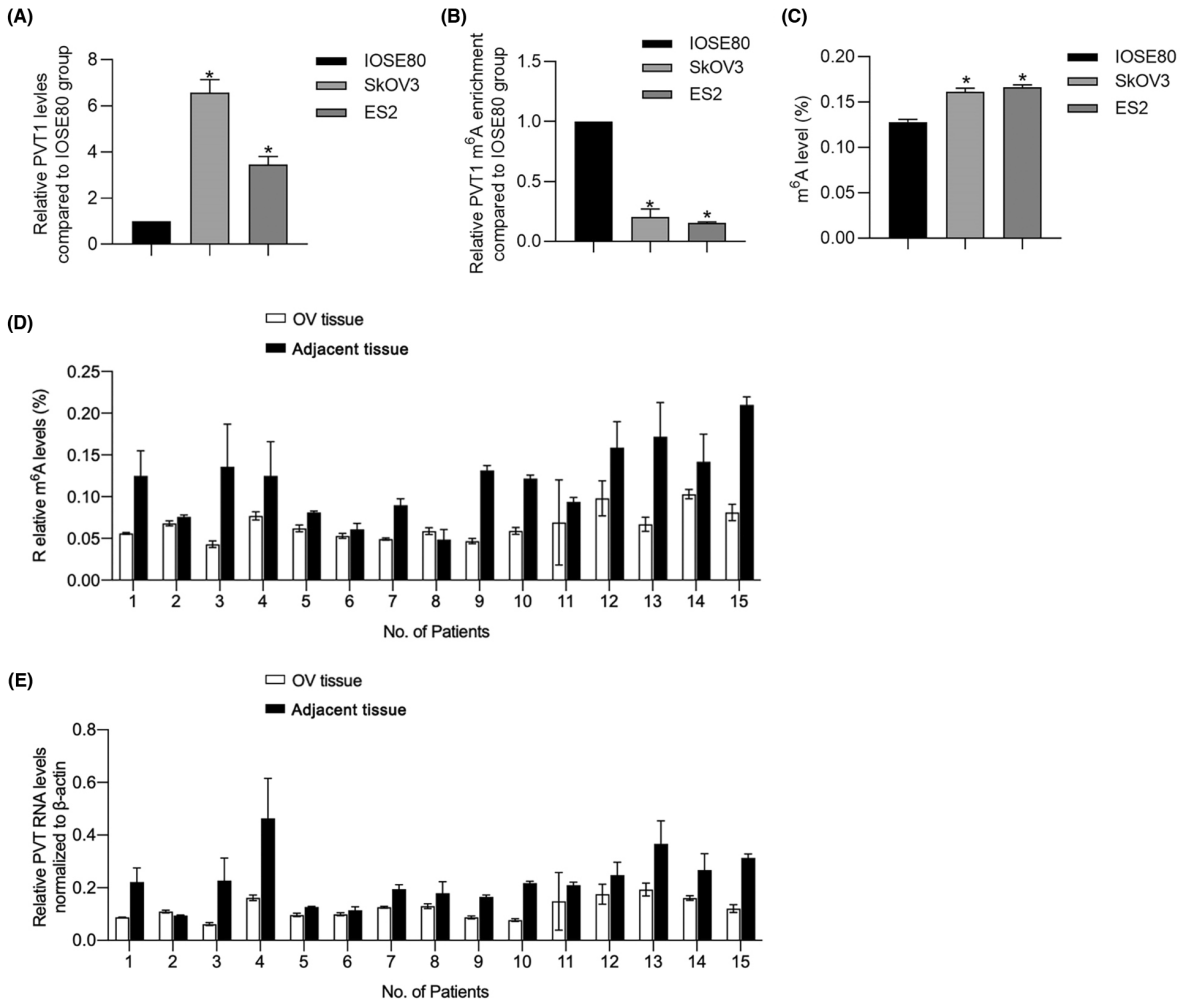
PVT1 RNA was demethylated in OV cells with relative high expression level. (A) RT‐qPCR was performed to detect PVT1 RNA levels in IOSE80, SkOV3 and ES2 cells. *p < 0.05, versus IOSE80 cells. (B) PVT1 methylation level was measured in IOSE80, SkOV3 and ES2 cells. *p < 0.05, versus IOSE80 cells. (C) global m^6^A methylation level was measured in IOSE80, SkOV3 and ES2 cells. *p < 0.05, versus IOSE80 cells. (D) global m^6^A methylation level was measured in 15 pairs of ovarian cancer tissues and adjacent normal tissues. (E) PVT1 RNA levels were detected by performing RT‐qPCR in 15 pairs of ovarian cancer tissues and adjacent normal tissues.

It is reported that, in osteosarcoma, ALKBH5‐mediated m^6^A demethylation of lncRNA PVT1 critically contributes to carcinogenesis via acting as an oncogene.^1^ This promoted us to detect m^6^A methylation level of PVT1 in OV cells. Expectedly, in OV cells, m^6^A methylation levels of PVT1 in both SkOV3 and ES2 cells were significantly lower than that in IOSE80 cells (Figure 1A). In our previous report,^2^ we reported that PVT1 in OV cells is upregulated than that in IOSE80 cells, which was further confirmed by performing RT‐qPCR (Figure 1B). These results indicated that PVT1 is significantly demethylated in OV cells. Meanwhile, we evaluated the total m^6^A methylation level in OV cells, notably, it is illustrated that total m^6^A methylation level of OV cells is significantly higher than that in IOSE80 cells (Figure 1C). To further confirm the correlation of m^6^A level with ovarian cancer progression, 15 pairs of OV tissues and adjacent normal tissues were collected for m^6^A level evaluation. As it is presented in Figure 1D, OV tissues presented significant higher m^6^A level, compared to adjacent normal tissues, which suggests that PVT1 methylation is specifically mediated, and may contributes to its roles in regulating malignancies in OV cells.

As reported previously,^19^ PVT1 exerts as an oncogene in OV, which promotes us to further detect relative PVT1 RNA in OV tissues compared to adjacent normal tissues. As presented in Figure 1E, PVT1 RNA is obviously higher than those in adjacent normal tissues (Figure 1E), indicating that PVT1 RNA levels present similar trend against global m^6^A methylation levels.

### 3.2 ALKBH5 is upregulated and caused PVT1 demethylation in OV cells

**FIGURE 2:**
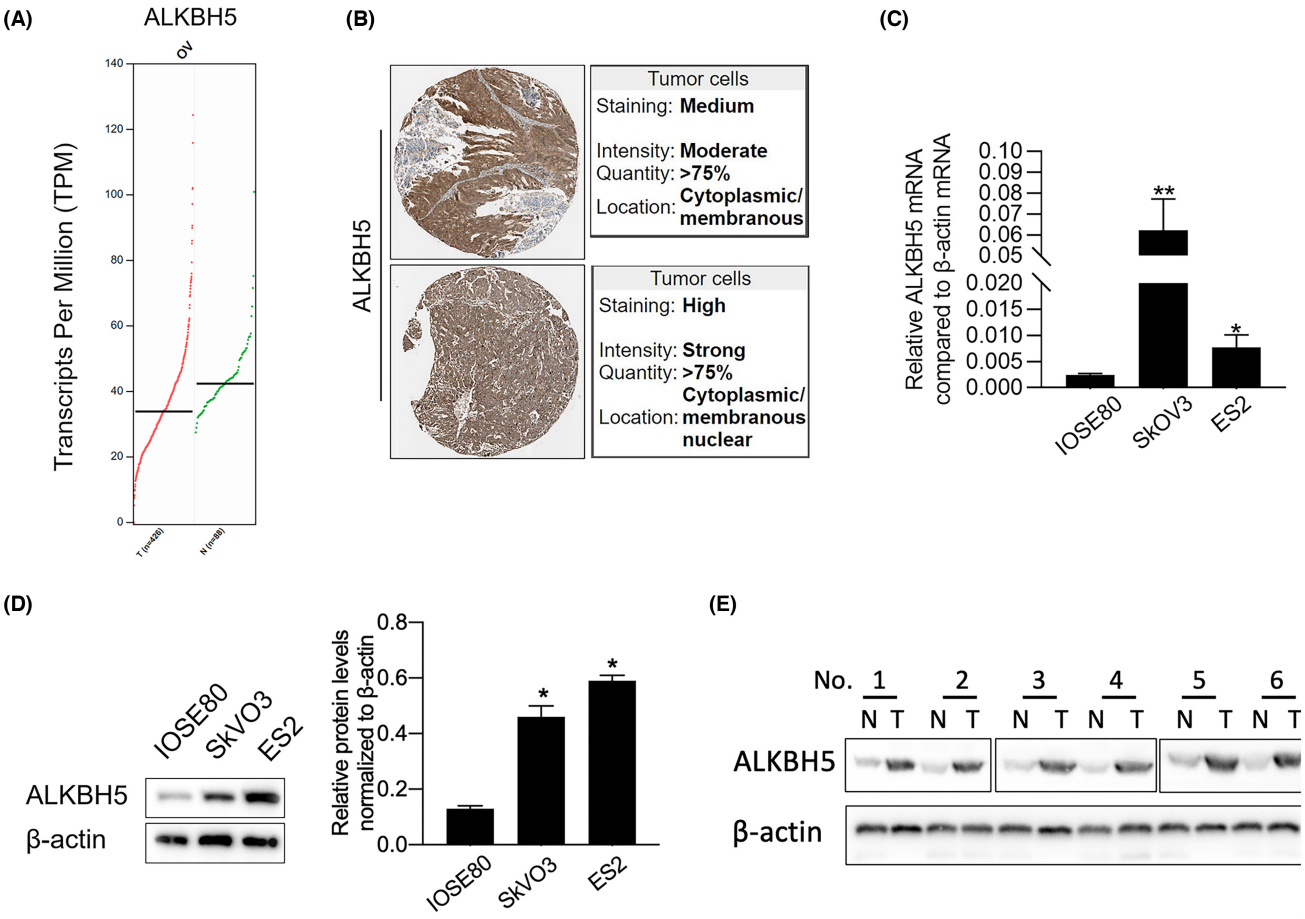
ALKBH5 expression was upregulated in OV cells. (A) the ALKBH5 mRNA levels in 426 OV tissues was compared with 88 normal tissues. (B) Human Protein Atlas was employed to detect ALKBH5 protein level in two OV tissues. To measure ALKBH5 mRNA and protein levels, qPCR (C) and Western blot (D) were performed (E) Western blot was performed to detect ALKBH5 in 6 pairs of non‐tumor (N) and tumor (T) tissues. *p < 0.05, versus IOSE80 cells, **p < 0.01, versus IOSE80 cells.

To verify whether ALKBH5 is abnormally expressed in OV tissue, transcriptional data and pathological protein expression were compared through GEPIA and Human Protein Atlas (HPA). As it is shown in Figure 2A,B, although ALKBH5 mRNA level in OV tissue was not obviously upregulated than that in adjacent tissues, OV tissue presented moderate and strong intensity in OV tissues. By performing RT‐qPCR and semiquantitative Western blot, it is obviously observed that in SkOV3 and ES2, ALKBH5 mRNA and protein levels were obviously upregulated than those in IOSE80 cells (Figure 2C,D). To further confirm whether ALKBH5 expresses differently in OV tissues compared to adjacent tissues, 15 pairs of OV tissues and adjacent normal tissues were employed to detect ALKBH5 protein level. Expectedly, ALKBH5 was obviously upregulated in OV tissues compared to adjacent tissues (Figure S1).

### 3.3 ALKBH5 physically binds to PVT1 RNA and tightly associated with PVT1 RNA presence

**FIGURE 3:**
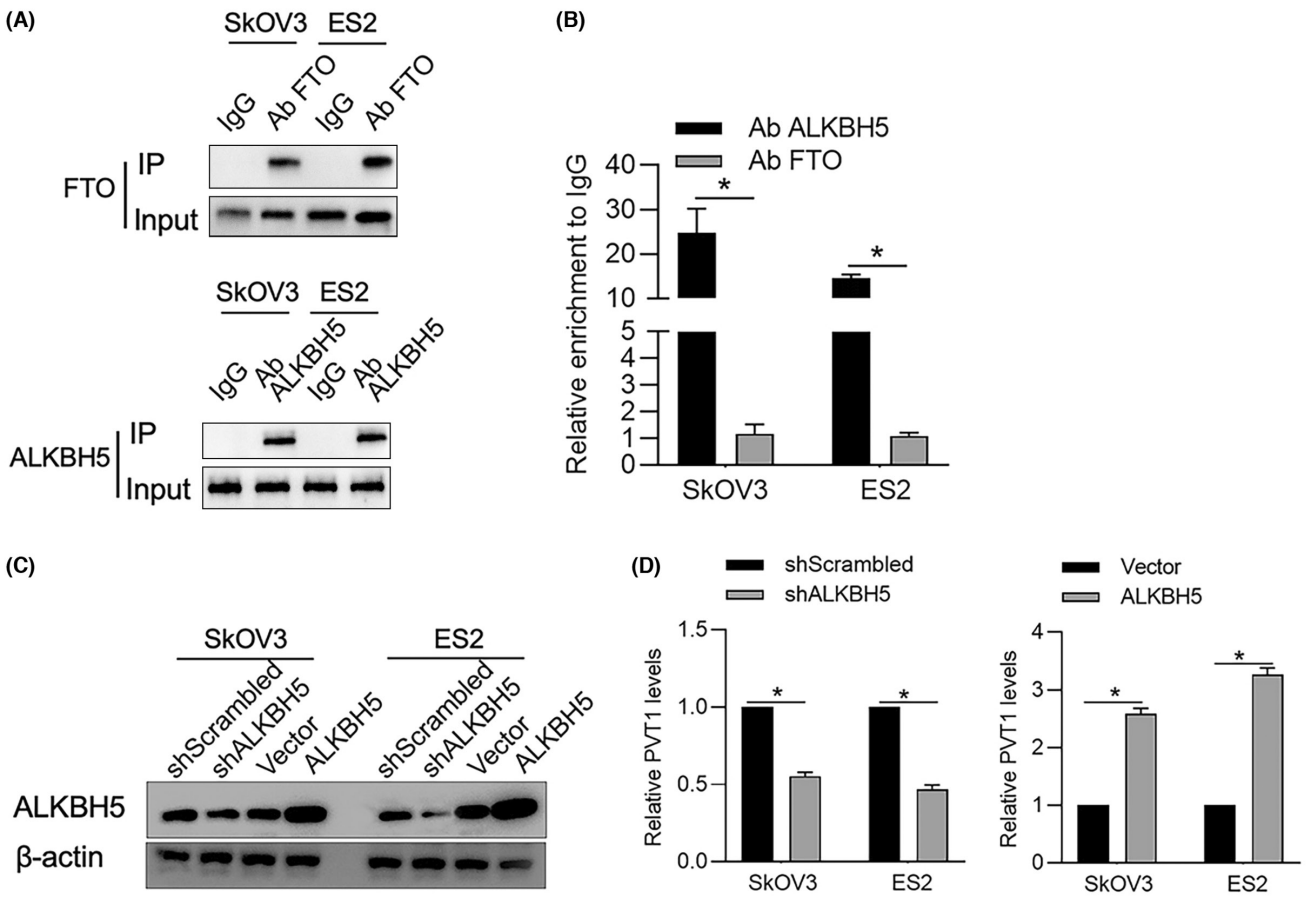
ALKBH5 physically binds to PVT1 RNA and maintains its stability. (A) ALKBH5 or FTO protein in immunoprecipitation product was detected by Western blot. (B) RT‐qPCR was performed to detect PVT1 RNA in immunoprecipitation product. *p < 0.05, versus Ab ALKBH5 group. (C) 48 h after ALKBH5 knockdown or overexpression, Western blot was measured to confirm the regulating efficiency. (D) RT‐qPCR was performed to detect PVT1 after ALKBH5 knockdown or overexpression. *p < 0.05, versus shScrambled group, or Vector group.

It is previously reported that, in osteosarcoma, ALKBH5, but not other m^6^A methylation regulating factors, tightly binds to PVT1 RNA and results in m^6^A demethylation of PVT1.^19^ This promoted us to confirm whether ALKBH5 and PVT1 physically interact in OV cells. To confirm this, RNA‐immunoprecipitation targeting to ALKBH5 followed by RT‐qPCR was performed. As it is shown in Figure 3A, both ALKBH5 and FTO, which was considered as a control, were efficiently immunoprecipitated in both SkOV3 and ES2 cells. PVT1 enrichment was significantly increased in immunoprecipitation product by using anti‐ALKBH5 antibody, but not in that of anti‐FTO antibody (Figure 3B), indicating that ALKBH5 directly binds to PVT1 RNA. By considering that ALKBH5 maintained PVT1 RNA level via inducing m^6^A demethylation,^20^ we then measured PVT1 levels after efficiently overexpression and knockdown of ALKBH5 (Figure 3C). Expectedly, knockdown of ALKBH5 significantly decreased PVT1 RNA in both two of OV cells, and overexpression of ALKBH5 significantly improved PVT1 RNA level (Figure 3D). All these results indicate that ALKBH5 may regulate PVT1 RNA via its demethylation activity.

### 3.4 ALKBH5 partially regulates FOXM1 via stabilizing PVT1 RNA

**FIGURE 4:**
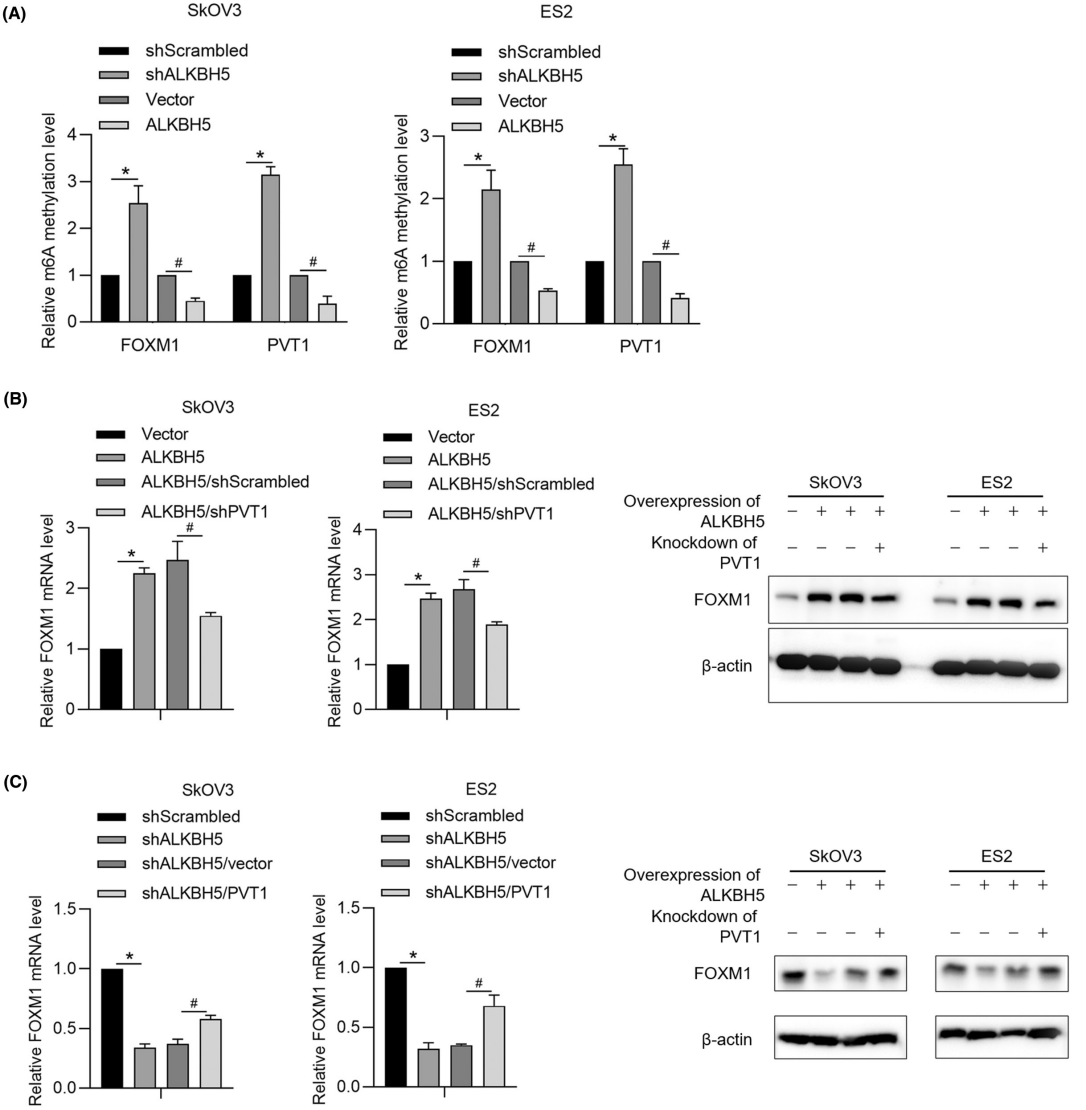
ALKBH5 partially regulates FOXM1 via PVT1. (A) After ALKBH5 knockdown or overexpression in SkOV3 and ES2 cells, methylation level of FOXM1 and PVT1 were measured. *p < 0.05, versus shScrambled group; #*p* < 0.05, versus vector group. (B) In ALKBH5‐overexpressed cells, the effect of PVT1 knockdown on FOXM1 mRNA and protein level were measured. *p < 0.05, versus vector group; #*p* < 0.05, versus ALKBH5/shScrambled group. (C) in ALKBH5‐knockdown cells, the effect of PVT1 overexpression on FOXM1 mRNA and protein level were measured. *p < 0.05, versus shScrambled group; #*p* < 0.05, versus shALKBH5/vector group.

In our previous report, we present that PVT1 epigenetically and post‐transcriptionally regulates FOXM1 by eliminating microRNA targeting to FOXM1 mRNA.^19^ Hao and colleagues found that, ALKBH5 mediates m^6^A demethylation of FOXM1 mRNA and promotes progression of uveal melanoma.^21^ This raises the question that whether ALKBH5 regulates FOXM1 indirectly via regulating PVT1 RNA. To confirm this, we overexpressed or knockdown ALKBH5 while modifying PVT1 RNA, and then detect FOXM1 mRNA and protein. Expectedly, overexpression or knockdown of ALKBH5 tightly regulates FOXM1 mRNA m^6^A methylation level and increased m^6^A methylation of PVT1 RNA (Figure 4A). To further confirm the specific regulation of PVT1 m^6^A methylation by ALKBH5 or FB23‐2, respectively, a specific inhibitor of FTO was employed to specifically inhibit FTO, but not ALKBH5, and ALKBH5 was knockdown by employing shRNA specifically targeting to ALKBH5 mRNA. Expectedly, as it is shown in Figure S2, addition of FB23‐2 remarkably increased global m^6^A methylation without obvious affection on PVT1 RNA m^6^A methylation, indicating that both ALKBH5 and FTO regulate m^6^A methylation of PVT1. Overexpressed ALKBH5 increased FOXM1 mRNA and protein, which were partially reversed by PVT1 knockdown (Figure 4B). Knockdown of ALKBH5 decreased FOXM1 mRNA and protein, which were partially reversed by PVT1 overexpression (Figure 4C).

### 3.5 Knockdown of ALKBH5 decreased OV malignancies which were partially reversed by PVT1 overexpression

**FIGURE 5:**
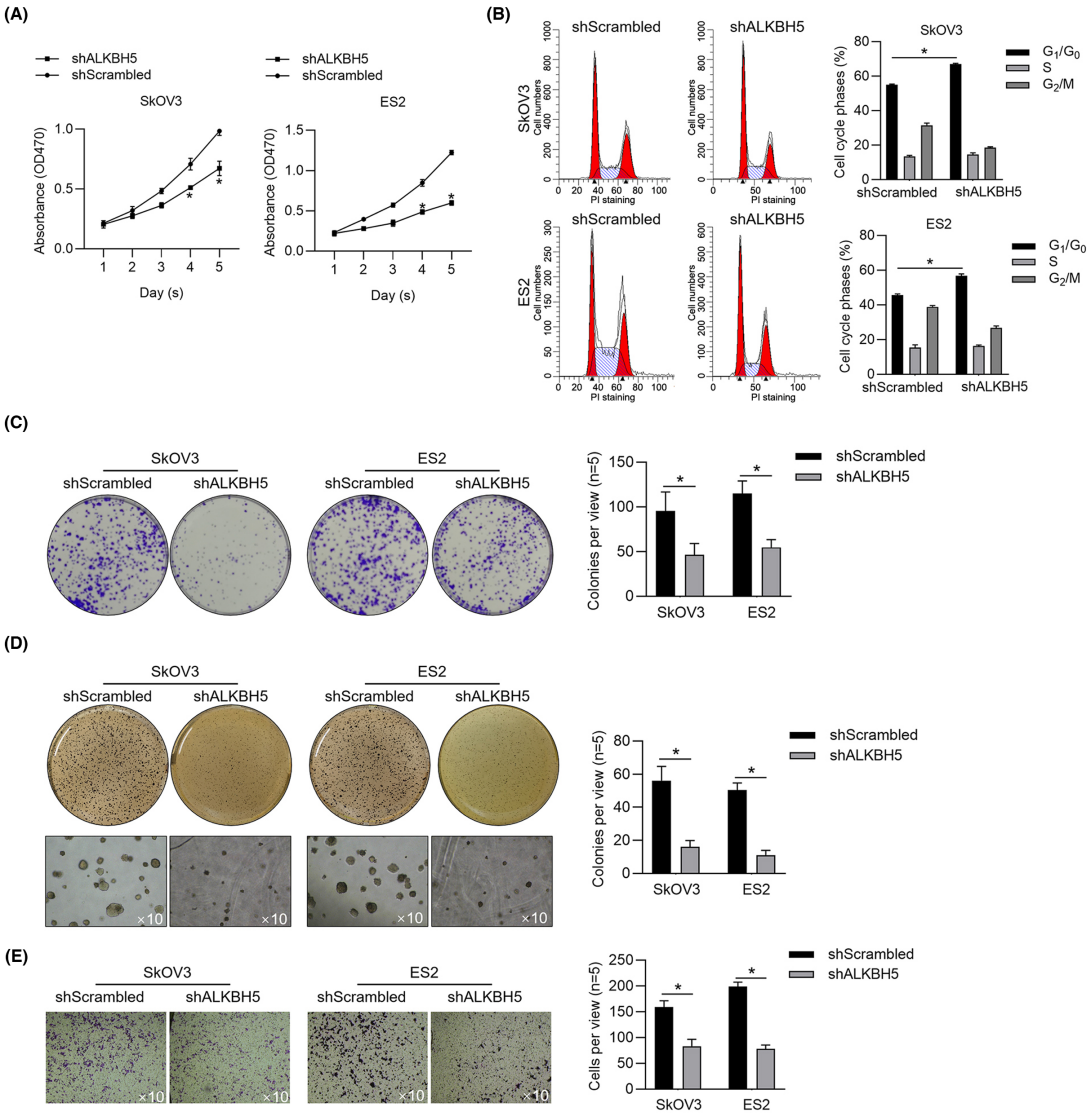
ALKBH5 knockdown decreased OV malignancies. (A) After ALKBH5 knockdown, cell viability was measured from day 1–5. *p<0.05, versus shScrambled group. After ALKBH5 knockdown, cell cycle distribution (B), colony formation (C), tumour formation in soft agar (D) and Transwell assay (E) were performed. *p < 0.05, versus shScrambled group.

By considering that ALKBH5 acts as oncogene in several kinds of cancer cells,^3^, ^4^, ^5^ we evaluate the effects of ALKBH5 knockdown on malignancies of OV cells, including SkOV3 and ES2. As it is shown that, knockdown of ALKBH5 significantly decreased cell viability from day 1 to 5 (Figure 5A), potentially via blocking cell cycle phases at G_1_/G_0_ (Figure 5B). We then measured the effects of ALKBH5 on colony formation, tumour formation and invasion, expectedly, it is presented that, knockdown of ALKBH5 significantly decreased all these phenomena (Figure 5C–E).

**FIGURE 6:**
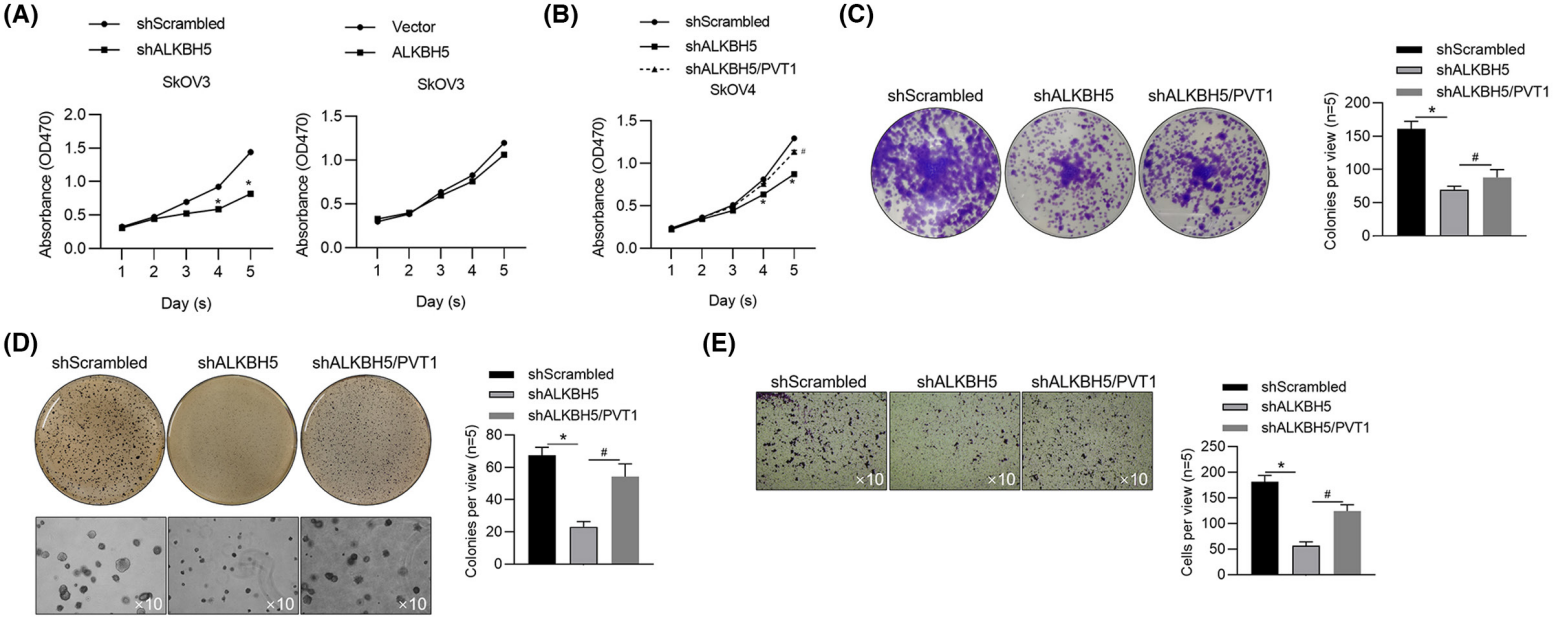
PVT1 overexpression partially reversed inhibition in malignant behaviours caused by ALKBH5 knockdown. (A) After ALKBH5 or PVT1 knockdown, cell viability from day 1 to 5 were measured by performing CCK‐8 assay. *p < 0.05, versus shScrambled group. In ALKBH5 stably knockdown cells, PVT1 was overexpressed and CCK‐8 assay was performed from day 1 to 5 (B), colony formation (C), tumour formation (D), and invasion ability (E) were also performed. *p < 0.05, versus shALKBH5 group.

To further confirm whether ALKBH5 affects malignant behaviours, at least partially, via regulating PVT1 RNA, we detected malignant behaviours affected by ALKBH5 with PVT1 knockdown. Before that, we evaluate the effect of ALKBH5 overexpression on cell viability, and found that although ALKBH5 knockdown decreased cell viability, ALKHB5 overexpression failed to obviously increase cell viability (Figure 6A), potentially due to high endogenous ALKBH5 level in SkOV3 cells. By considering this, we knocked down ALKHB5 and then overexpressed PVT1 in SkOV3. As it is shown in Figure 6B–D, ALKBH5 knockdown decreased cell viability, colony formation, tumour formation in soft agar and invasion, expectedly, partially reversed by PVT1 overexpression, indicating that ALKBH5 affects OV partially via regulating PVT1 methylation.

### 3.6 ALKBH5 knockdown increased chemosensitivity via regulating PVT1

Previously, we reported that PVT1 epigenetically maintains FOXM1 protein level,^19^ which is protein characterized by its role in inducing sensitization to antitumor drugs in cancer cells.^20^, ^21^, ^22^ By considering that PVT1 was recently reported to be a enhancer of chemosensitivity in cancer cells, we used several agents with different mechanisms of action: direct DNA damage (carboplatin), DNA synthesis inhibition (5‐FU) or cell division disruption (paclitaxel).

**FIGURE 7:**
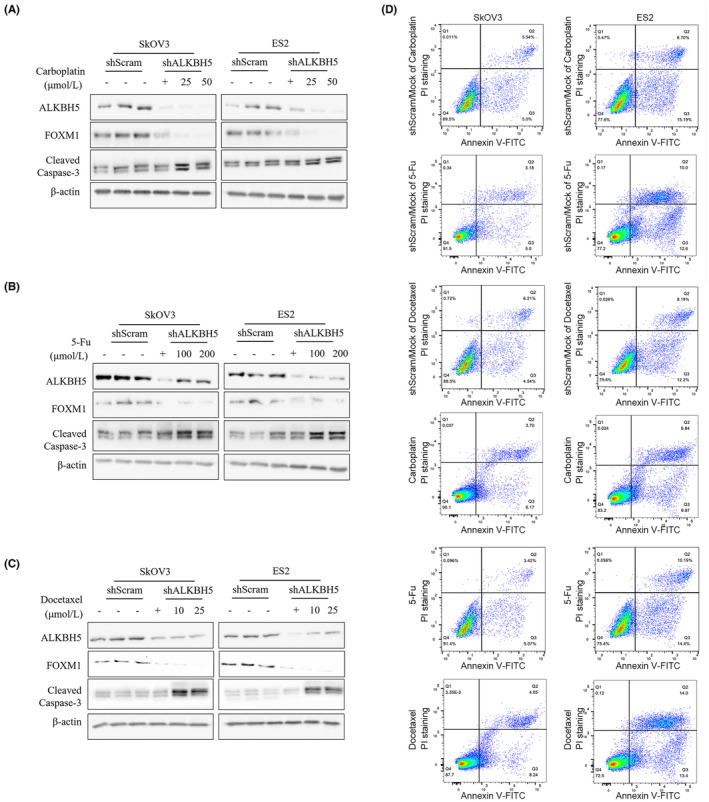
ALKHB5 knockdown sensitize OV cells to Carboplatin, 5‐Fu and Docetaxel. After Carboplatin, 5‐Fu and Docetaxel treatment, ALKHB5, FOXM1, cleaved caspase‐3 were measured by Western blot (A, B, C). (D) After 50 μmol/L of Carboplatin, 200 μmol/L of 5‐Fu or 25 μmol/L of Docetaxel treatment, apoptosis rate was measured by performing Annexin V‐FITC/PI double staining followed by flow cytometry analysis.

Consistent with previous result, ALKHB5 knockdown intensively decreased FOXM1 protein level, without obviously affects cleaved caspase‐3 (Figure 7A–C). In shScrambled‐transfected groups, Carboplatin, 5‐Fu or Docetaxel treatments slightly affected cleaved caspase‐3. After ALKBH5 knockdown groups, Carboplatin, 5‐Fu or Docetaxel treatment obviously increased cleaved‐capase3 levels. To further confirm, whether ALKBH5 knockdown sensitize cells to Carboplatin, 5‐Fu or Docetaxel treatment, 50 μmol/L of Carboplatin, 200 μmol/L of 5‐Fu or 25 μmol/L of Docetaxel treatment was picked for treatment followed by apoptosis detection by Annexin V‐FITC/PI staining. Expectedly, after ALKBH5 knockdown, Carboplatin, 5‐Fu or Docetaxel treatment obviously increased cell proportion of FITC+/PI+ and FITC+/PI‐ (Figure 7D). By considering that ALKBH5 regulates FOXM1 mainly via PVT1 (Figure 4B,C), it is concluded that ALKHB5 regulates chemosensitivity in OV cells via regulating PVT1.

### 3.7 ALKBH5 promotes OV development in nude mice

**FIGURE 8:**
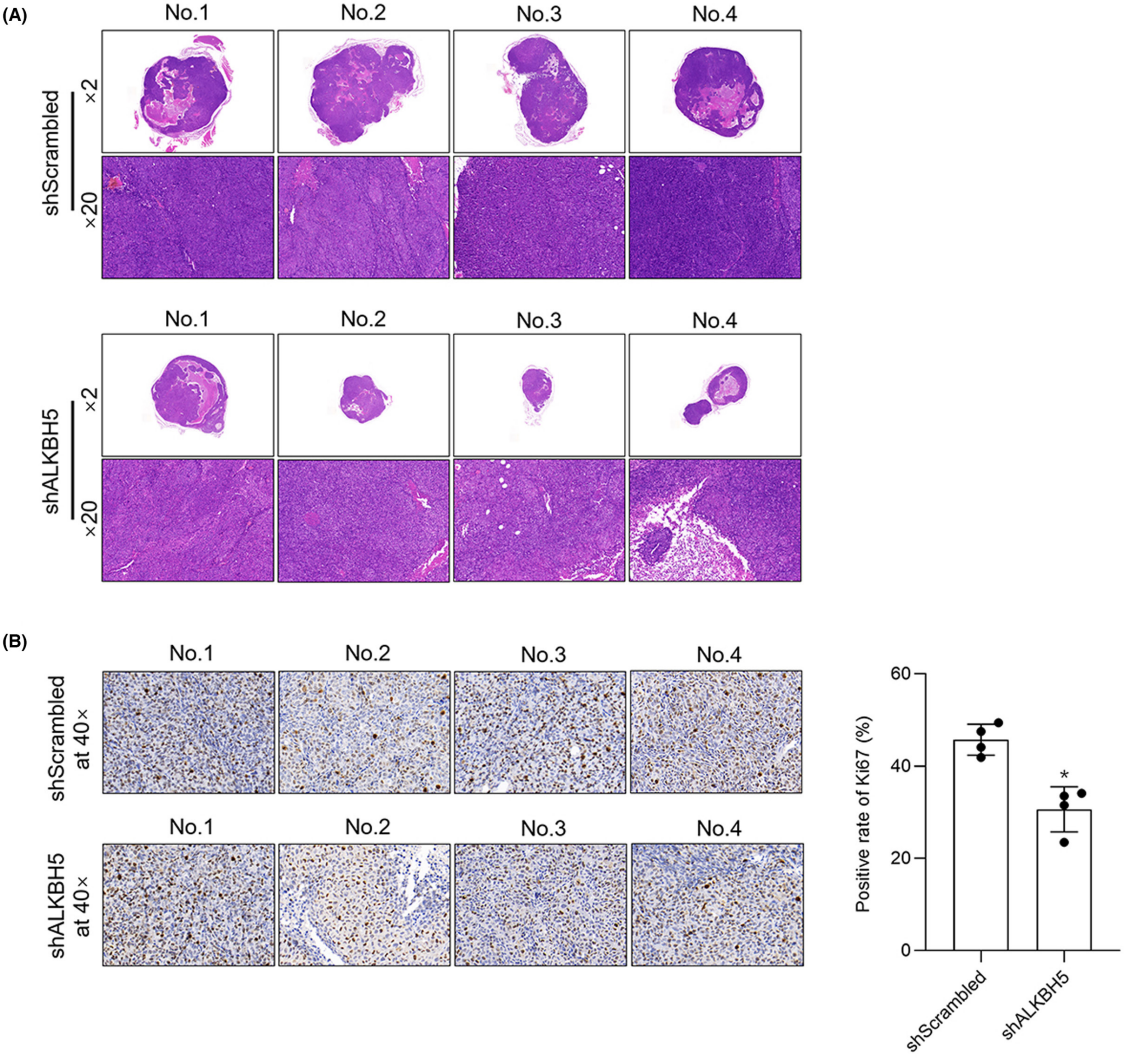
ALKBH5 knockdown impacted tumour growth in vivo. (A) ALKBH5 knockdown inhibited the growth of SkOV3 cell tumours (*n* = 4). (B) Staining of Ki67 was presented. *p < 0.05, versus shScram group.

After partially ascertaining the oncogene roles of ALKHB5 via regulating PVT1 in OV, its potential roles as a therapeutic target in OV was then investigated. A tumour xenograft model was established by subcutaneously injecting ALKBH5‐knockdown SkOV3 cells into nude mice. As it is shown in Figure 8A, ALKBH5 knockdown impaired the growth of SkOV3 cell tumours. To detect the effects of ALKBH5 knockdown on tumours, Ki67 staining was performed. As predicted, ALKBH5 knockdown decreased positive proportion of Ki67 (Figure 8B), indicating that ALKBH5 knockdown impaired tumour growth via inhibiting cell proliferation. Although it is still unidentified whether PVT1 is critical in tumour growth, our results demonstrated that ALKBH5 might regulates tumour progression via PVT1 RNA.

## 4 DISCUSSION

In our present study, we demonstrated that ALKBH5, which is a critical RNA demethylase, is posttranscriptionally overexpressed in ovarian cancer and related cell lines. Upregulated ALKBH5 demethylated the long noncoding RNA PVT1 and thus stabilized FOXM1, at least partially, in ovarian cancer. Recently, accumulating evidence has shown that m^6^A methylation, which is considered the most common mRNA internal modification in higher eukaryotes,^9^, ^10^ tightly regulates ovarian malignancies via m^6^A methyltransferases or demethylases, including METTL3, METTL14 and WTAP.^11^, ^12^, ^13^, ^14^, ^15^ This study reveals a novel regulatory mechanism of ALKBH5 in ovarian cancer: it modifies PVT1 RNA and subsequently stabilizes FOXM1.

LncRNA PVT1 plays a regulatory role in gynaecological cancer and is involved in regulating many processes of cellular biology, such as the proliferation, migration, invasion and drug resistance of tumour cells.^6^ It can interact with miRNAs, mRNAs and related proteins, which have different effects in different tumours. In most tumours, it has a carcinogenic effect, and in a few tumours, it plays a role as a tumour suppressor gene. In addition, in studies of ovarian cancer, lncRNA PVT1 not only participates in various physiological processes of tumour cells but also mediates related signal transmission and pathological changes through several mechanisms, such as epigenetic modification and molecular sponging, which may be a new target for the treatment of ovarian cancer.^19^ In addition, the differences in the function of lncRNA PVT1 in different studies may be caused by the diversity of therapeutic drugs and cancer cell lines. Therefore, it is necessary to further clarify the effect and mechanism of lncRNA PVT1 on ovarian cancer in many samples and a variety of ovarian cancer cell lines. Previously, we reported that in ovarian cancer, PVT1 acts as a microRNA sponge and subsequently regulates FOXM1.^19^ Hao and colleagues reported that ALKBH5‐mediated m^6^A demethylation of FOXM1 mRNA promotes malignancies of uveal melanoma,^23^ which demonstrated that ALKBH5 increased FOXM1 stability by demethylating FOXM1 mRNA. This prompted us to confirm whether ALKBH5 regulates FOXM1 via PVT1 RNA in ovarian cancer. According to our results, knockdown of ALKBH5 decreased FOXM1 protein, which was partially reversed by overexpression of PVT1, indicating that ALKBH5 regulates FOXM1 partially via PVT1 RNA.

Methylation of m^6^A is a double‐edged sword because excessive modification or abnormally low modification levels may lead to the occurrence and progression of tumours. m^6^A methylation‐related enzymes can be used as regulatory targets of lncRNAs and miRNAs, thus affecting the m^6^A modification level of other RNAs, which is a research idea that can be explored. Moreover, the same m^6^A methylation‐related enzyme may have different functions in different pathways, suggesting that there may be multiple targets. In addition, m^6^A methylases may have other independent functions in addition to regulating m^6^A levels. For instance, METTL3 can enhance the translation of cancer‐related genes, in addition to its role as a methyltransferase, and can affect cancer progression independently of its catalytic subunit^1^; thus, these findings can be used to expand m^6^A research.

Much progress has been made in the study of the role of m^6^A modification in ovarian cancer, but its specific role is still controversial. Whether m^6^A and its regulatory factors can be used as potential biomarkers for the diagnosis and prognosis evaluation of ovarian cancer and its specificity and sensitivity still need to be explored. Some studies have shown that m^6^A regulatory factors and related pathways can be used as therapeutic targets, but there is no specific application in large‐sample clinical practice, so its side effects are not clear to a large extent. In addition, whether there is a joint effect or a subtractive effect between m^6^A modification and other epigenetic modifications needs to be further explored. ALKBH5 is tightly involved in the biological regulation of many cancers, such as ovarian cancer,^24^ colon cancer,^25^, ^26^ pancreatic cancer^27^ and gastric cancer.^28^ Surprisingly, in ovarian cancer tissues, the ALKBH5 expression level was similar to that in adjacent nontumor tissues, and its protein level was significantly higher in ovarian cancer tissues than in adjacent tissues. This indicated that ALKBH5 may be posttranscriptionally regulated in various cancer types and thus results in its controversial effects.

## 5 CONCLUSION

In conclusion, we demonstrate that ALKBH5 promotes tumour progression by at least partially stabilizing PVT1 and FOXM1 in ovarian cancer. Therefore, ALKBH5 is a promising target for ovarian cancer molecular therapy.

## AUTHOR CONTRIBUTIONS

**Lin Li:** Data curation (equal); investigation (lead); methodology (lead); software (equal); validation (equal); writing – original draft (lead). **Jie Chen:** Conceptualization (supporting); data curation (equal); investigation (supporting); project administration (lead); validation (lead). **Ao Wang:** Investigation (supporting); writing – original draft (supporting); writing – review and editing (equal). **Ke Yi:** Conceptualization (lead); formal analysis (lead); funding acquisition (lead); methodology (lead); resources (equal); supervision (lead); writing – original draft (equal).

## FUNDING INFORMATION

This study was supported by the Key research and development Subjects of Sichuan Province (grant no. 2022YFS0079).

## CONFLICT OF INTEREST STATEMENT

No conflicts of interests was declared.

## Supporting information


Figure S1

Figure S2

## Data Availability

All data generated or analysed during this study are included in this published article.
