# Supplementary material for: ALKBH5 regulates ovarian cancer growth via demethylating long noncoding RNA PVT1 in ovarian cancer
Source: J Cell Mol Med. 2023 Dec 14;28(2):e18066. doi: 10.1111/jcmm.18066 (PMC10826426; doi:10.1111/jcmm.18066)
Supplement: Supplementary file 1 — Figure S1 [file JCMM-28-e18066-s001.zip › Supplementary Figure 1.docx]

**Supplementary Figure** **1. ALKBH5 expression in ovarian cancer tissues.**
15 pairs of ovarian cancer tissues and adjacent tissues were employed to detect ALKBH5 protein levels by performing western blot.
