# Supplementary figures and images for: ALKBH5 regulates ovarian cancer growth via demethylating long noncoding RNA PVT1 in ovarian cancer
Source: J Cell Mol Med. 2023 Dec 14;28(2):e18066. doi: 10.1111/jcmm.18066 (PMC10826426; doi:10.1111/jcmm.18066)

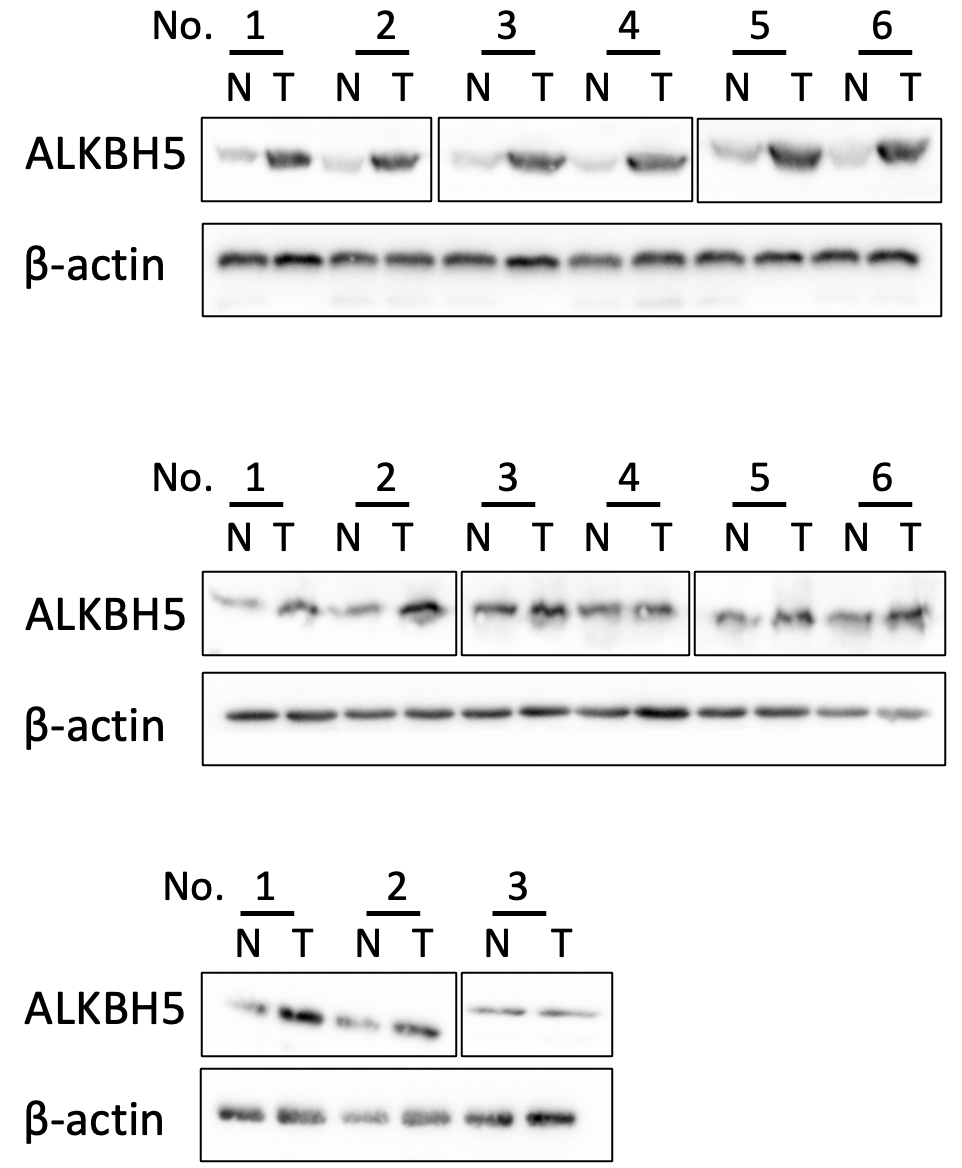

Supplement: Supplementary file 1 — Figure S1 [file JCMM-28-e18066-s001.zip › jcmm18066-sup-0001-FigureS1.jpg]

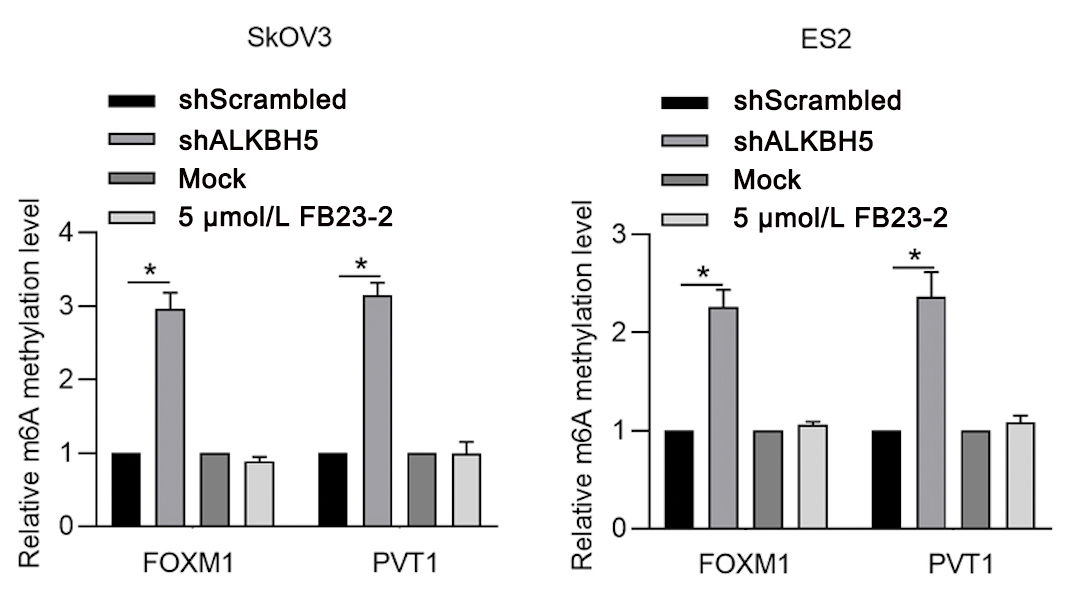

Supplement: Supplementary file 2 — Figure S2 [file JCMM-28-e18066-s002.zip › jcmm18066-sup-0002-FigureS2.jpg]
