# Supplementary material for: ALKBH5 regulates ovarian cancer growth via demethylating long noncoding RNA PVT1 in ovarian cancer
Source: J Cell Mol Med. 2023 Dec 14;28(2):e18066. doi: 10.1111/jcmm.18066 (PMC10826426; doi:10.1111/jcmm.18066)
Supplement: Supplementary file 2 — Figure S2 [file JCMM-28-e18066-s002.zip › Supplementary Figure 2.docx]

**Supplementary Figure** **2. FOXM1 and PVT1 m^6^A methylation**
By ALKBH5 knockdown or addition of FB23-2, FOXM1 and PVT1 RNA m^6^A methylation are detected.
